# Expression of Concern: MicroRNA-26a reduces synovial inflammation and cartilage injury in osteoarthritis of knee joints through impairing the NF-κB signaling pathway

**DOI:** 10.1042/BSR-2018-2025_EOC

**Published:** 2022-05-30

**Keywords:** Cartilage injury, Inflammatory injury, MicroRNA-26a, NF-κB signaling pathway, Osteoarthritis, Synovial inflammation

The Editorial Office has been made aware of potential issues surrounding the scientific validity of this paper, including duplications between the BMS-345541 panel of Figure 6D and the miR-26a mimics panel of figure 3D, one of which appears to be vertically flipped and the colour altered, as well as duplications found between cells on the Figure 4C miR-26a mimic panel and the Figure 7C BMS-345541 panel, which has also been vertically flipped. The authors have been contacted with regards to these concerns but have currently not responded to the Journal's queries.

